# Transforming medical education in Liberia through an international community of inquiry

**DOI:** 10.1371/journal.pgph.0001610

**Published:** 2023-03-08

**Authors:** Kristina Talbert-Slagle, Ibrahim Ajami, Braden Currey, Rachel Galvao, Jerusalem Hadush, Serene Silin Li, Javaughn T. Flowers, Moses Ziah, Desmond Amuh, Mikaela Rabb, Olayinka Stephen Ilesanmi, Nikole Allen, Marie Martin, Mary Miller, Attila Yaman, Tej Nuthulaganti, Chelsea Plyler, Odell Kumeh, Joseph Sieka, Onyema Ogbuagu, Regan Marsh, Asghar Rastegar, Lawrence Sherman, Z’Sherman Adams, Angela Benson, Bernice Dahn

**Affiliations:** 1 Department of Medicine, Yale University, New Haven, Connecticut, United States of America; 2 Republic of Liberia Ministry of Health, Monrovia, Liberia; 3 Liberian College of Physicians and Surgeons, Monrovia, Liberia; 4 Department of Health Policy, Vanderbilt University, Nashville, Tennessee, United States of America; 5 Clinton Health Access Initiative, Boston, Massachusetts, United States of America; 6 University of Liberia College of Health Sciences, Monrovia, Liberia; 7 Department of Emergency Medicine, Brigham and Women’s Hospital, Boston, Massachusetts, United States of America; 8 A.M. Dogliotti School of Medicine, University of Liberia College of Health Sciences, Monrovia, Liberia; Institute of Public Health Bengaluru, INDIA

## Abstract

A critical component of building capacity in Liberia’s physician workforce involves strengthening the country’s only medical school, A.M. Dogliotti School of Medicine. Beginning in 2015, senior health sector stakeholders in Liberia invited faculty and staff from U.S. academic institutions and non-governmental organizations to partner with them on improving undergraduate medical education in Liberia. Over the subsequent six years, the members of this partnership came together through an iterative, mutual-learning process and created what William Torbert et al describe as a “community of inquiry,” in which practitioners and researchers pair action and inquiry toward evidence-informed practice and organizational transformation. Incorporating faculty, practitioners, and students from Liberia and the U.S., the community of inquiry consistently focused on following the vision, goals, and priorities of leadership in Liberia, irrespective of funding source or institutional affiliation. The work of the community of inquiry has incorporated multiple mixed methods assessments, stakeholder discussions, strategic planning, and collaborative self-reflection, resulting in transformation of medical education in Liberia. We suggest that the community of inquiry approach reported here can serve as a model for others seeking to form sustainable global health partnerships focused on organizational transformation.

## Introduction

### Medical education in Liberia

Liberia’s health system made great strides in the decade following its 14 years of civil war (1989–2003). President Ellen Johnson Sirleaf’s leadership, along with collaborative international partnerships, generated national improvements in several key health indicators from 2004–2014. Liberia met Millennium Development Goal #4 to reduce childhood mortality by two-thirds ahead of the global target dates [1, 2]. However, the emergence of Ebola in Liberia in 2014 stymied progress in the health sector and revealed ongoing fragilities in the health system [3, 4]. Inadequacies in Liberia’s health workforce, including one of the lowest physician-to-population ratios worldwide, were exacerbated by Ebola’s devastating toll: 8% of the country’s physicians, nurses, and midwives were killed by the virus [5]. As of 2015, Liberia had just 80 physicians providing services for a population of over four million [6].

In response to the Ebola crisis, the Ministry of Health of Liberia developed the Investment Plan for Building a Resilient Health System post-Ebola. Launched in 2015, the Investment Plan was accompanied by a national Health Workforce Strategy, which built on insights from Rwanda’s Human Resources for Health program [7, 8], and focused on targeted increases in the quantity, quality, and skill diversity of Liberia’s public sector health workforce [6, 9].

A critical component of strengthening Liberia’s physician workforce centered around Liberia’s only medical school, A.M. Dogliotti School of Medicine (AMD). Founded in 1968 in the capital city of Monrovia and now housed within the College of Health Sciences at the University of Liberia, AMD was once considered a model for medical training in West Africa, with a post-secondary, seven-year educational program that required two years of premedical instruction followed by five years of clinical instruction [10]. However, the country’s 14-year civil war caused looting or closure of more than 80% of its government health facilities, prompting most medical faculty to leave the country and leaving 90% of the country’s population without access to health care [11, 12]. AMD consequently experienced severe, ongoing shortages of faculty and other resources, both during and after the war, with concomitant damage to the quality of all levels of education, including undergraduate medical education, and significant reductions in numbers of medical school graduates. The 2014–15 Ebola crisis further jeopardized post-war recovery at AMD, causing temporary closure of the school and claiming the lives of several medical faculty members and students [13].

After Liberia’s civil war ended in 2003, additional requirements were added for M.D. training to compensate for weaknesses in the country’s secondary school education, as described by the Dean of the medical school and other medical school leadership. [oral communication, October 2016] At the time that this partnership began in 2015, only students who had completed a four-year Bachelor’s of Science degree (B.Sc.) were eligible to enroll in AMD’s five-year medical school, pending completion of other admission requirements. Obtaining an M.D. in Liberia therefore required 9 years of instruction (4 years B.Sc. plus 5 years M.D.) after secondary school. This model differed from that of other medical schools in the West African region that required six years to complete a medical degree post-secondary school, including in Sierra Leone [14], Nigeria [15], and Ghana [16, 17]. Despite these changes, AMD continued to experience high rates of attrition, often with fewer than half of initially-enrolled students graduating with an M.D., according to medical school students and leadership [oral communication, October 2016–2017] leaving the government of Liberia unable to meet its targets for a fit-for-purpose physician workforce [6].

This manuscript describes a six-year process of organizational transformation, in which Liberian faculty members and students came together with a group of international partners to completely restructure and reform M.D. education in Liberia. The decision to transform medical education was not made lightly, nor all at once. Rather, it emerged through an action-inquiry process, through an interwoven series of empirical analyses, stakeholder discussions, training programs, and working group meetings, in the process generating a highly collaborative partnership in which researchers and practitioners learned with and from each other. Both the research findings and the ways that they were used to effect organizational transformation are described here, framed within an approach to social science research called a “Community of Inquiry.” [18, 19]

## Methods

### The “community of inquiry” approach

Cooperative Ecological Inquiry has been described by William Torbert [18] as an approach to social science in which all participants are focused on “creating real-time communities of inquiry that bridge subjectivities and differences of perspective and support peaceful, ecologically-sensitive personal and organizational transformation.” Torbert has also described collaborative inquiry as “consider[ing] the researcher to be an interactive participant, rather than a detached observer…and would welcome the possibility of change through dialog between the actor-researcher and others” [19] and a process wherein “each actor can gain increasingly valid knowledge of social situations only as other actors collaborate in inquiry, disclosing their being, testing their knowledge, discovering shared purposes, and producing preferred outcomes.”

Collectively, these descriptions of collaborative inquiry and “real-time communities of inquiry” best capture the approach to learning, discovery, and organizational transformational processes and outcomes that occurred at AMD from 2015–2022. This community of inquiry approach to social science research can incorporate multiple social science research methods but most importantly hinges on a collaborative, mutual-learning effort involving inquiry and action by researchers and practitioners with multiple, different perspectives. The action-inquiry process reported here includes three studies conducted at AMD and in the larger physician community in Liberia in 2016, 2017, and 2018, as well as multiple stakeholder and practitioner engagement meetings, convened by Liberia’s most senior UL, medical and health leadership from 2017–2021, in order to ensure that findings from the mixed methods studies were used for evidence-based decision-making.

The three studies reported here were conceptualized and initiated by Liberian leaders and collaboratively designed and implemented by international academic partners and Liberian partners. The international researcher-participants in this community of inquiry learned how better to understand, interpret, and apply the data to improving medical education in Liberia by engaging in this collaborative action-inquiry process with their Liberian partners, and vice versa. This manuscript therefore includes not only social science approaches to surveys and interviews, but also the stakeholder engagements and decision-making that occurred within and between data collection and analysis, which collectively generated organizational transformation at AMD.

### 2016 survey and interviews

To develop the initial research study in 2016, a Yale faculty member and a group of Yale undergraduate students collaborated with Liberia’s Minister of Health and Minister of Education and a Liberian medical student appointed by the Minister of Health to adapt a validated survey instrument used by the Association of American Medical Colleges to fit the Liberian context, with the goal of identifying current challenges at AMD [20]. At the time the survey was distributed, the medical school was on break, and many students had left campus. Informed consent forms and surveys were distributed in person at a convening of medical students who had remained on campus. The objectives of the study were presented by the Yale faculty member, followed by a question-and-answer session. Students who elected to complete the paper survey were given informed consent forms and then the survey, itself. Surveys were also distributed digitally via a link emailed to all AMD students by a fifth-year AMD medical student leader who was a member of the research team. Most respondents completed the paper survey.

Survey respondents’ answers were compiled as summary statistics, followed by an examination of the tie-adjusted Spearman rank correlation between responses to questions and whether students had considered dropping out and/or had ever wondered about whether they would graduate. This is a nonparametric method that does not rely on standard assumptions of normality because we have ordinal Likert scale data. The significance of each correlation value was tested at the standard p = 0.05 level. All data analysis was conducted using the R software package version 3.3.2.

An open-ended interview guide was also developed by the research team, including the same Yale faculty member, the Minister of Health, and students from Yale and AMD. The interview guide was utilized to direct conversations with AMD students and faculty. Interviews ranged in duration from 20 to 100 minutes and were conducted in English, the official language of Liberia. Recorded interviews were transcribed. Some interviews were conducted by a mixed pairing of U.S. and Liberian interviewers; others were conducted only by U.S. interviewers. In all cases, research team members (from the U.S. and Liberia) met frequently to discuss and debrief the interview experiences, and transcripts were reviewed against audio by Liberian team members to ensure accurate transcription, accounting for local dialects and accents. Interviews were conducted until the research team determined that theoretical saturation had been reached [21, 22].

Individual interviews were conducted with eighteen medical students. Respondents were recruited for interviews either because they signed up to be interviewed after the initial question-and-answer session or were named by interview respondents via a snowball sampling approach and subsequently agreed to be interviewed. The research team also facilitated an additional focus group discussion with medical and pharmacy students who share the same campus and classrooms and have some of the same introductory courses.

The research team used thematic analysis to identify emergent themes [21, 22]. Upon reading all of the transcripts, a preliminary list of codes was proposed by a member of the research team and utilized for initial coding of 3 transcripts. The code list was then adapted with input from a Yale faculty member with expertise in qualitative research. Three members of the research team collectively updated the code list and developed sub-codes. Next, the three members independently applied the final code list to the remaining transcripts, deliberated over each transcript, resolved differences through negotiated consensus, and identified emergent themes from excerpts exported from Dedoose version 7.5.6 (UCLA, U.S.A.).

### 2017 survey

To develop a more comprehensive understanding of financial needs and challenges for students at AMD as well as factors to consider for long-term financial sustainability for AMD, the research team developed a 20-question survey exploring basic personal financial information. The research team included faculty and students from both the University of Liberia and Yale University. Surveys were distributed in paper format by a fifth-year medical student to who was a member of the research team, and respondents’ answers were entered into Microsoft Excel and compiled as summary statistics.

### Institutional review board exemption for 2016–17 surveys and 2016 interviews

The Yale University Institutional Review Board (IRB) Human Subjects Committee deemed both the 2016 and 2017 studies exempt from IRB review (HSC #1610018485) in October 2016 and (IRB #2000021882) in October 2017. Despite the exemption determinations, all members of the research teams completed Human Subjects Research Protection training prior to the start of data collection. Verbal or check-box informed consent was obtained prior to each survey.

### 2018 survey and institutional review board approval

To explore the professional knowledge, experience, goals, and self-assessed competencies [23] of 5^th^-year medical students, AMD alumni, and other practicing physicians in and around Liberia, U.S. academic faculty developed a survey instrument, which was reviewed and revised by AMD faculty and administrators. Survey responses were offered on a five-point Likert scale. A separate section of the survey presented the current AMD curriculum, asking AMD alumni to assess its relevance to training future physicians for the practice of medicine and to determine whether each course had been delivered in a manner that AMD alumni felt was either adequate or inadequate. This survey was developed to capture a wide range of data related to physician training and pipeline development in Liberia. Results relevant to undergraduate medical education and curriculum revision at AMD are presented here.

A research team comprised of Liberian and U.S. faculty and staff administered the survey on paper to 124 respondents, including final-year medical students and physicians at various stages of training, such as interns (who are immediate past graduates of AMD), residents, and faculty, and then inputted the survey data into REDCap electronic data capture tools hosted at Vanderbilt University [24, 25]. REDCap (Research Electronic Data Capture) is a secure, web-based software platform designed to support data capture for research studies. Data are presented as summary statistics. Survey respondents were recruited via email from the Dean of AMD as well as a senior official at the Liberian Ministry of Health who sent a digital survey link to all physicians in Liberia working in government health facilities. No responses were received through the digital survey link. All responses were received via paper surveys distributed by the research team in and around the nation’s capital, which represents the most densely-populated urban area of the country.

The Yale University Institutional Review Board declared the study (Protocol # 2000024198) exempt from review because it was considered a quality improvement study and not human research. An independent institutional review board in Liberia known as UL-PIRE IRB and the Vanderbilt University Institutional Review Board (IRB #181899) concurred with the exemption determination. Despite the exemption determination, all members of the research team completed Human Subjects Research Protection training prior to the start of data collection, and verbal or check-box informed consent was obtained prior to each survey and interview.

## Results

**Fig 1:**
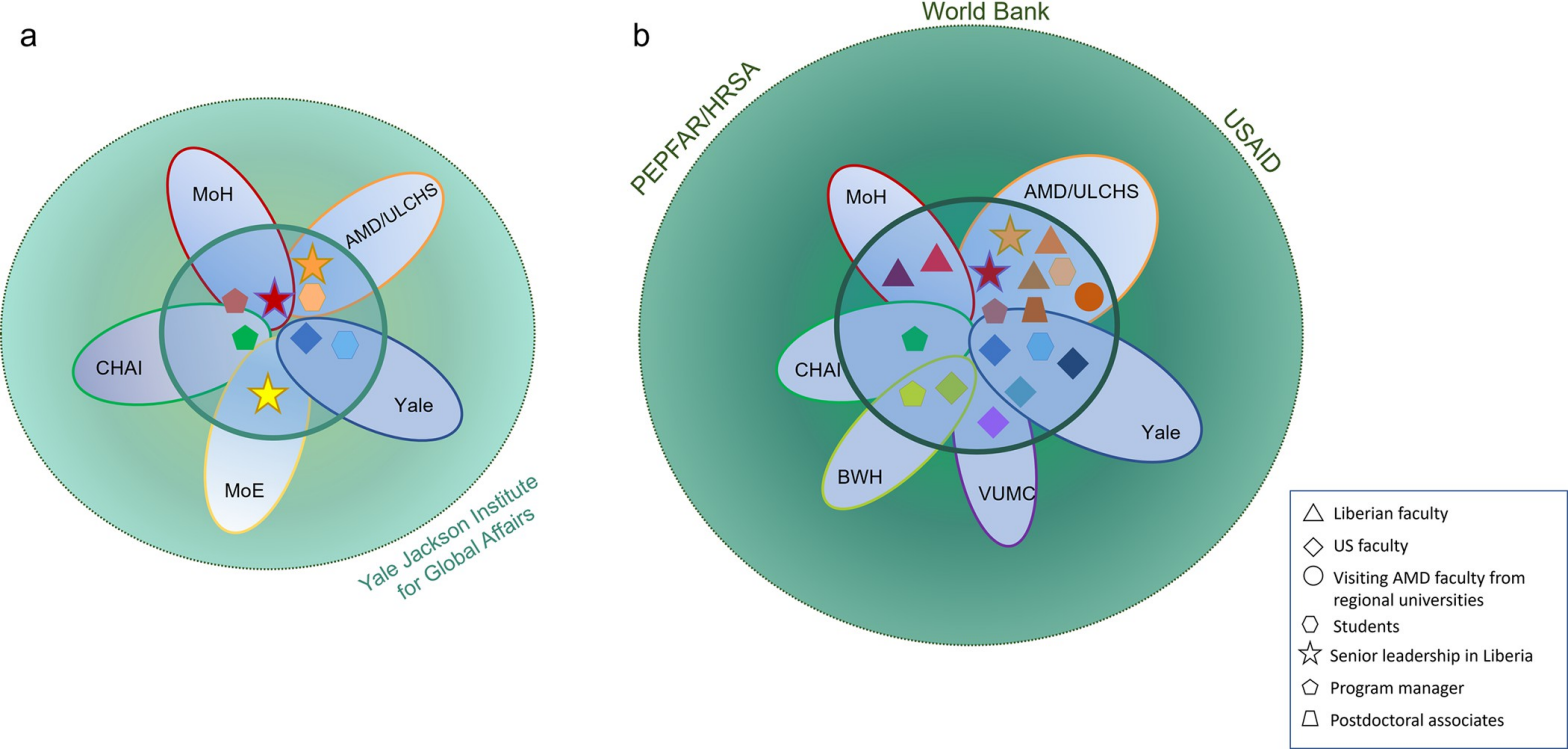
Formation of a community of inquiry through an international action-inquiry partnership. This figure depicts the individuals, institutions, and groups [26] involved in this community of inquiry after it first came together in 2015 at the direction of the Minister of Health of Liberia to support the country’s Health Workforce Program Strategy. a) In 2016, the President of Liberia asked the Ministers of Health (MoH) and Education (MoE) to improve conditions for medical students at Liberia’s only medical school, A.M. Dogliotti School of Medicine (AMD), which is part of the University of Liberia College of Health Sciences (ULCHS). The Ministers commissioned a mixed methods study from a Yale University (Yale) faculty member and her students, in collaboration with the Dean of AMD, and with facilitative partnership from program managers at the Clinton Health Access Initiative, Inc. (CHAI). The action and inquiry steps pursued by this team founded the community of inquiry, with funding from the Yale Jackson Institute for Global Affairs and, later PEPFAR/HRSA. b) The community of inquiry expanded in subsequent years to include additional faculty and students at AMD, including visiting AMD faculty from west and east African medical schools, as well as faculty, staff, and program managers from Yale, Vanderbilt University Medical Center (VUMC), Brigham and Women’s Hospital (BWH), and Liberia’s Ministry of Health, with distinct (and sometimes intermittent) funding from a variety of sources (rendered complementary by the efforts of the community of inquiry) including the World Bank, USAID, and PEPFAR/HRSA.

Fig 1 indicates the institutions and roles of individuals involved in formation of the community of inquiry, with early members in 2016 (Fig 1A) and the more expanded community of inquiry, which had formed as of 2018 (Fig 1B). The community of inquiry is depicted by the smaller green circle in both figures. Initially, the community of inquiry built upon the foundations formed by existing partnerships and close collaborative relationships between the Ministries of Health and Education of Liberia and the Clinton Health Access Initiative, Inc (CHAI), which provided technical assistance for the Health Workforce Strategy [6], and between Yale University and the Ministry of Health, who had worked together on health workforce capacity building in health management prior to the Ebola crisis, as shown in Fig 1A. The community of inquiry formed gradually, with some fluidity as students and trainees entered and exited the group, but all members of the community of inquiry shared a few key characteristics: sustained commitment to following the vision and guidance of the Liberian leadership, willingness to share knowledge and admit lack of knowledge, eagerness to learn from each other’s perspectives, expertise, and experiences, and interest in bridging research and practice toward accomplishing long-term goals set by Liberian members of the group.

The first research study conducted as part of this action-inquiry partnership was initiated in 2016 by a request from the Minister of Health to explore needs and challenges experienced by medical students at AMD. A mixed methods study was developed by the initial members of the community of inquiry (Fig 1A), including a survey exploring students’ experiences with faculty, housing and other university infrastructure, and finances related to their medical education. A discussion guide was also developed to explore these same topics in more detail. Sixty-nine students began the survey and 61 students fully completed it out of a total of 219 students enrolled at AMD, for a response rate of 27.9%. Forty-three percent (26/61) of the respondents were preclinical students in years 1–2 of AMD, and the remaining 57 percent (35/61) were in years 3–5. No other demographic data was collected. Individual interviews were conducted with 18 medical students, and a focus group discussion was also conducted with pharmacy and medical students.

Thematic analysis of the qualitative data generated three emergent themes: demoralizing learning environment, deteriorated infrastructure, and financial challenges, which were further substantiated by the quantitative findings.

The demoralizing learning environment was attributable to challenges with faculty, including faculty shortages, harsh teaching approaches, and high failure rates. The Dean of AMD indicated that the medical school needed a minimum of 41 faculty members to teach in both preclinical (14 faculty needed, minimum) and clinical (27 faculty needed, minimum) departments. At the time of the 2016 study, only 22 people were available to teach in total: 9 in the preclinical sciences, and 13 in the clinical sciences (S1 Table). Although shortages of clinical faculty posed difficulties, efforts to include students in patient rounds and teaching on-site at the university teaching hospital enabled many clinicians to provide at least some clinical instruction to third- through fifth-year medical students. The severe dearth of preclinical faculty, however, caused ongoing deficits in teaching and learning, with solutions often involving short-term “fly-in, fly-out” instruction from foreign faculty, or simply the lack of certain preclinical instruction altogether. The impact of these faculty shortages on student learning and morale were profound, as the representative quotes in Table 1 indicate.

**Table 1:** Impact of faculty shortages on AMD student learning (2016).

Respondent	Representative Quote
**Student**	“We don’t have enough lectures, like there are times in semester like we don’t do certain courses [be]cause there is no lecturer to teach that course, so I don’t think we get any like what medical education should be. I think they are just trying to make it work. [Be]cause we need doctors and we have to train doctors. But as of right now. . . I don’t think the school is at a point where we are actually provided with quality medical education.” (Student 12)
**Faculty**	“Before the Liberian civil war, we trained a lot of people and they left from here and went to Britain and the United States and never came back, so if you look around you will find very few graduates of AM Dogliotti College of Medicine—there are not many and that is because all those who were trained in the 1980’s left the country.” (Faculty 2)
**Focus group (students 17 and 18)**	***Student 18*:** “Sometimes we will call, we will call the professor. He will say ‘Oh I will come. You guys just wait on me. In the next thirty minutes, I will be there.’ Every day, in the next thirty minutes, he will be there, and he would not reach. And then that week would end. And another week will begin.” ***Interviewer*:** “Why do you think that is? What’s going on behind the scenes there?” ***Student 17*:** “Because they are doing other jobs.”
**Student**	When a visiting professor comes to teach a course… they give you that 2 weeks, we have to come for Monday to Friday and that course will be covered in that 2 weeks period of time. So you have over 1000 slides that you have to read in that two weeks and be able to comprehend before you can go to do an exam. So at times, it’s very difficult to remember all of those slides in that two weeks and go for the exam. So these are some of the problems that we facing.” (Student 1)

**Fig 2:**
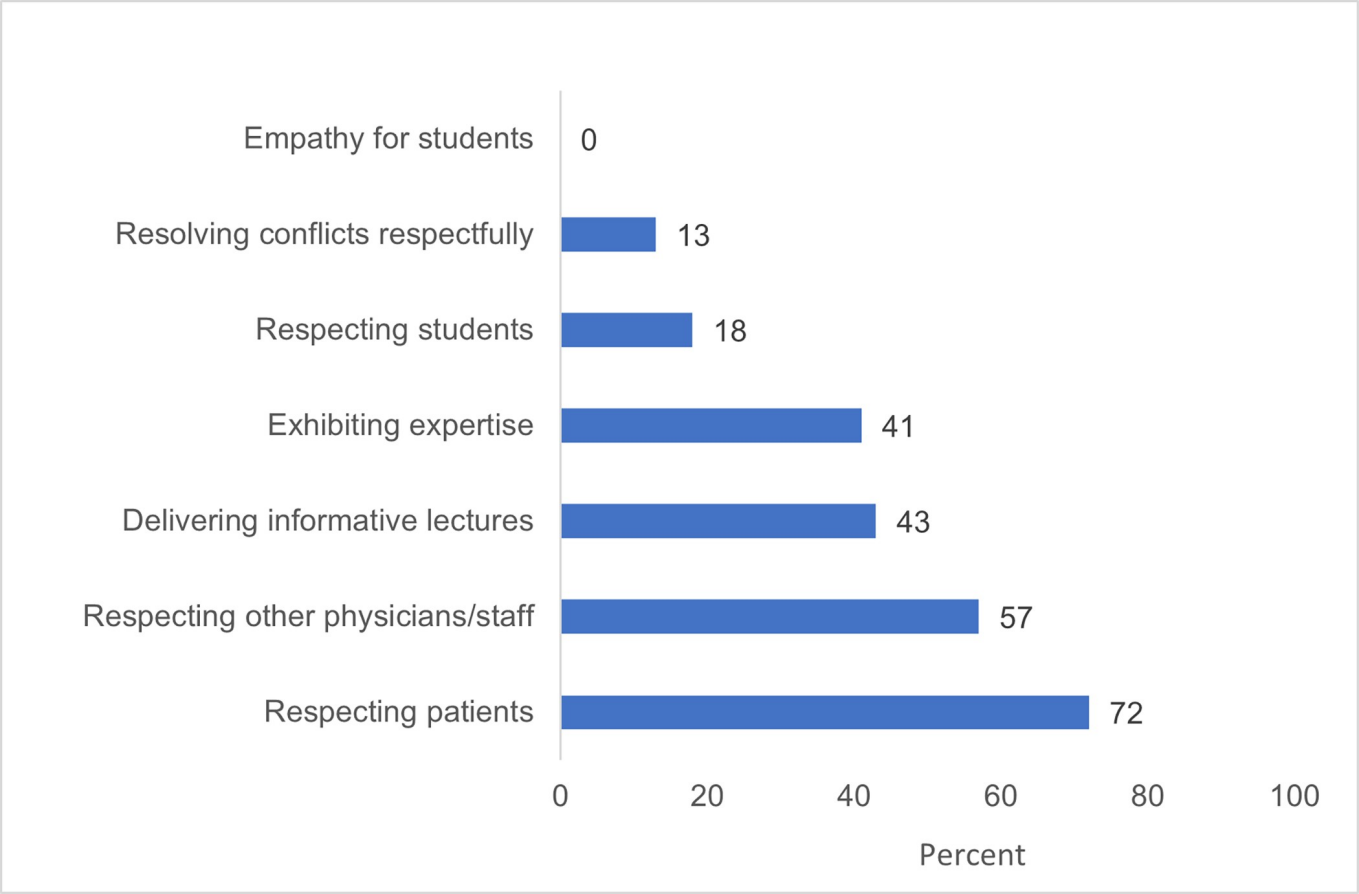
Percentage of AMD students who thought faculty display the following behaviors “most of the time” or “always” (2016).

Students also reported ineffective or harsh teaching tactics, and lack of empathy, engagement with, and mentorship from faculty, (Fig 2 and Table 2), as well as high levels of exam failure and fear of failure (Table 3).

**Table 2:** Student reports of ineffective teaching tactics (2016).

Respondent	Representative Quote
**Student**	“It’s just like not caring enough to like make sure that the students are getting what they are trying to at the class” (Student 12)
**Student**	“If you ask questions, well trust me, you either get insulted in the process, or even before answering your questions, he will make you look really stupid for asking it.” (Student 11)
**Student**	“Students aren’t being guided on what to do. Not every department, but few professors, few of them do that. And then I think that’s the only reason why students fail. Not that they are dull, but you have a bunch of the things to read and you are not being guided on what to read.” (Student 9)
**Student**	“He will just go online, look for someone’s powerpoint, bring it and then he will refuse to use the projector, or because he doesn’t want us to know that it’s someone else’s work that he is using, and he starts reading.” (Student 2)

**Table 3:** Student experiences with medical school exams (2016).

Respondent	Representative Quote
**Student**	“I mean, how are we to prepare for that? You give me A, B, C to study, and time of the examination you give me I, J, K, L, what do you think I will do. . . Yeah but for sure, if you teach the lesson, and you bring questions from that lesson, we will pass. And what makes it worse is that they will not show up, like most of them, as I told you they will not show up until exams are around the corner. Ok, so that alone poses stress on the students.” (Student 9)
**Student**	“You cannot be free to express yourself with professors, you are not free, professors come, sometime he doesn’t even give you paper to see it, he just put grade up and you cannot challenge it, you cannot say why do I have this? Why is that? They do not give papers at time, they just put grades up. You see it, we pass, if you don’t pass, that’s it, you have to accept it.” (Student 3)
**Student**	“We live in fear more than we live in any other thing. Every day you think they will terminate your contract tomorrow. . . Everybody is sitting on the edge. Am I going to do a re-sit? And it’s such that, for the school of medicine preclinical years, you are given one chance. Meaning, if you have five courses, and you happen to pass four but fail one, you will go home and rest for a semester and come and join those who will be starting freshly. Ok? And to come from that situation and to rise up is demeaning. If you fail all, they tell you, pack your bag and go, this place is not for you. . . Somebody can go as far as fifth year, they will deny you and then drop you. What becomes of such a person?. . .If I can fight on my own and reach third year, you’ve got to produce me as a doctor. So, your dropping issue should be somewhere around first [year]. . .because the person just started so they would not mind. But having gone three years, you still want to drop me? To go and do what?” (Student 17)

**Fig 3:**
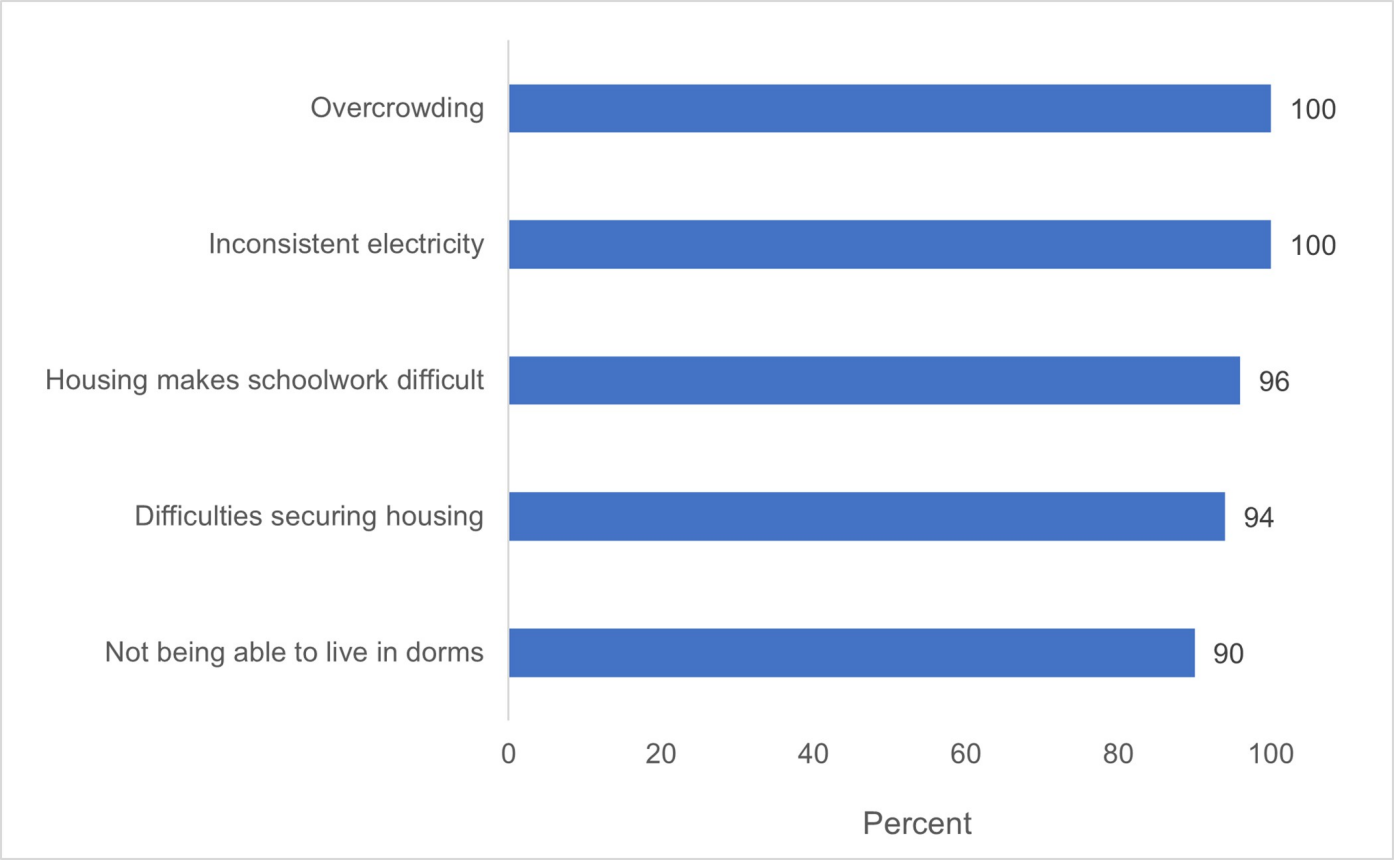
Percentage of students who experienced the following conditions in medical school dorms (2016).

Deteriorated infrastructure at the medical school included dormitories (Fig 3) and classrooms. From survey data, sixty-five percent of respondents agreed with the statement “The facilities at AMD are a barrier to my success.” A resource-mapping exercise indicated that there were too few classrooms for the number of medical students and that available rooms were often run-down, damaged, or poorly equipped, eg with broken projectors or inadequate air conditioning. Students reported that the medical library had outdated books, no internet, and inconvenient hours, closing at 4 pm. Students further reported that internet was completely unavailable in most areas of campus, which the AMD Dean confirmed. Representative quotes describing deteriorated infrastructure are shown in Table 4.

**Table 4:** AMD students’ experiences with medical school infrastructure (2016).

Respondent	Representative Quote
**Student**	“You got four to five students in each room. And the worst of it all, during examinations, it can go as far as eight persons in one single room.” (Student 17)
**Student**	“It is crowded and sometimes it’s even difficult in getting ready to go to the hospital in the morning because we all have to use that same bathroom so the time that it would take for everyone to get through. Honestly some people even end up taking bath outside just so they can get ready in time to be there.” (Student 2)
**Student**	“The dormitories? One looks so bad, even our friends when they come, they will see us, and be like ‘wow, is it the kind of building for living for medical school?’ So, for me, I, whenever the wind is strong, I just feel, many times I feel that the building might collapse.” (Student 11)
**Student**	“So I don’t want to reach for something, you know like even electricity just recently became stable. We were living in darkness, we now have electricity on campus. Thank God we have now. Even the Internet facility, I don’t want to even go there, because that’s not even my concern now. Once we have the basic things like provide more rooms for students, provide professors, provide material you need to work with, ok we think about the internet, we think about the other things.” (Student 9)
**Student**	“We don’t have water running, it has to come from the hand pump to draw water, there is no drinking water on campus, only hand pump, and the hand pump is not even chlorified, so before you drink water you have to boil it.” (Student 3)

Student financial challenges emerged as another major theme from both quantitative and qualitative data in the 2016 study. Since medical school is supposed to be full-time, AMD students are not allowed to work and are supposed to receive a government stipend of 200 US dollars per month to cover living expenses. Students widely reported in 2016 that stipends were not paid on time, sometimes with multiple-month delays. Ninety-four percent of respondents agreed with the statement, “I have faced significant financial challenges while at AMD,” and 75% agreed with the statement, “I worry that I will not have enough money to buy food.” Many students also reported having to approach family for financial support and/or being unable to afford basic necessities. Representative quotes describing the emergent theme of financial challenges are shown in Table 5.

**Table 5:** AMD students’ financial challenges (2016).

Respondent	Representative Quote
**Student**	“You have to go back home, begging family members, you know, to help you. And you know, this is a second degree that we’re running after we got B.S. In Liberia once you have B.S. people expect you to start working and helping your family, so being there all the time, you know, begging, ‘please help me, please help me,’ so it can be very embarrassing. But we can just be forced to go back and say ‘you just have to do it for us because we are stranded. There’s nothing we can do.’” (Student 6)
**Student**	“Our second term, the stipend was an issue, the university said they were out of money, and you notice that the failure rate increased because students were not concentrating.” (Student 13)
**Student**	“You don’t have money to buy the books. To even buy the books… you cannot find it around here. Even to buy it online you should have the money. The money is not even there to buy it online. So to get the books and even read is very hard.” (Student 4)

Several specific issues that emerged within the themes of demoralizing learning environment, deteriorated infrastructure, and financial challenges correlated significantly (p < 0.05) with students’ having considered dropping out of medical school and/or wondering if they would graduate (Table 6). Given that attrition at AMD was known to be high, the significant relationship between students’ having considered dropping out of medical school and feeling that faculty are not generally helpful (p<0.01), agreeing that facilities are a barrier to success (p<0.01), and worrying that they cannot buy food (p = 0.01) or would be a financial burden on family (p<0.01) indicated potential areas for additional attention or intervention to improve medical students’ success (and retention) at AMD. Several of these same factors were significantly associated with students’ wondering whether they would graduate from medical school, including if faculty were considered unhelpful (p = 0.03) or displaying inappropriate behavior (p = 0.02), as well as feeling that the facilities were a barrier to success (p = 0.03), overcrowding in dorms (p = 0.01), or having difficulty getting housing in dorms (p = 0.03). Students who agreed that housing negatively affected their schoolwork were also significantly more likely to wonder if they would graduate (p<0.01). As with considering dropping out, students’ worrying that they would not be able to buy food (p = 0.04), as well as worrying that they would be a financial burden on family were both significantly associated with students’ wondering whether they would graduate (p = 0.03). Collectively, these results indicate that challenges with faculty, infrastructure, and finances increased students’ past (“have considered dropping out”) and future ("wonder about graduating”) worries about whether or not they would complete medical school.

**Table 6:** Relationship between AMD medical students’ experiences and their concerns about completing medical school (2016).

		Considered dropping out		Wonder about graduating	
		correlation	p-value	correlation	p-value
**Demoralizing learning environment**	Faculty are generally helpful	-0.38**	<0.01*	-0.28	0.03*
	Faculty help those having difficulty	-0.21	0.10	-0.18	0.18
	Faculty are overly distant	-0.04	0.76	0.25	0.05
	Faculty display inappropriate behavior	0.23	0.07	0.29	0.02*
**Deteriorated infrastructure**	Facilities are a barrier to my success	0.41	<0.01*	0.28	0.03*
	Overcrowding	0.09	0.55	0.39	0.01*
	Inconsistent electricity	0.23	0.11	0.14	0.33
	Difficult to get housing in dorms	0.06	0.66	0.31	0.03*
	Housing negatively impacts schoolwork	0.22	0.12	0.42	<0.01*
**Financial challenges**	Students face financial challenges	0.23	0.07	0.23	0.07
	Worry that I cannot buy food	0.32	0.01*	0.27	0.04*
	Worry that I cannot buy personal items	0.25	0.06	0.22	0.09
	Worry that I cannot buy school supplies	0.21	0.10	0.22	0.08
	Financial burden on family	0.37	<0.01*	0.28	0.03*

*p-value <0.05, considered statistically significant

**Negative correlation values indicate inverse effect (e.g. students who answered “disagree or strongly disagree” that faculty were generally helpful were significantly more likely to have considered dropping out or wonder about graduating).

**Fig 4:**
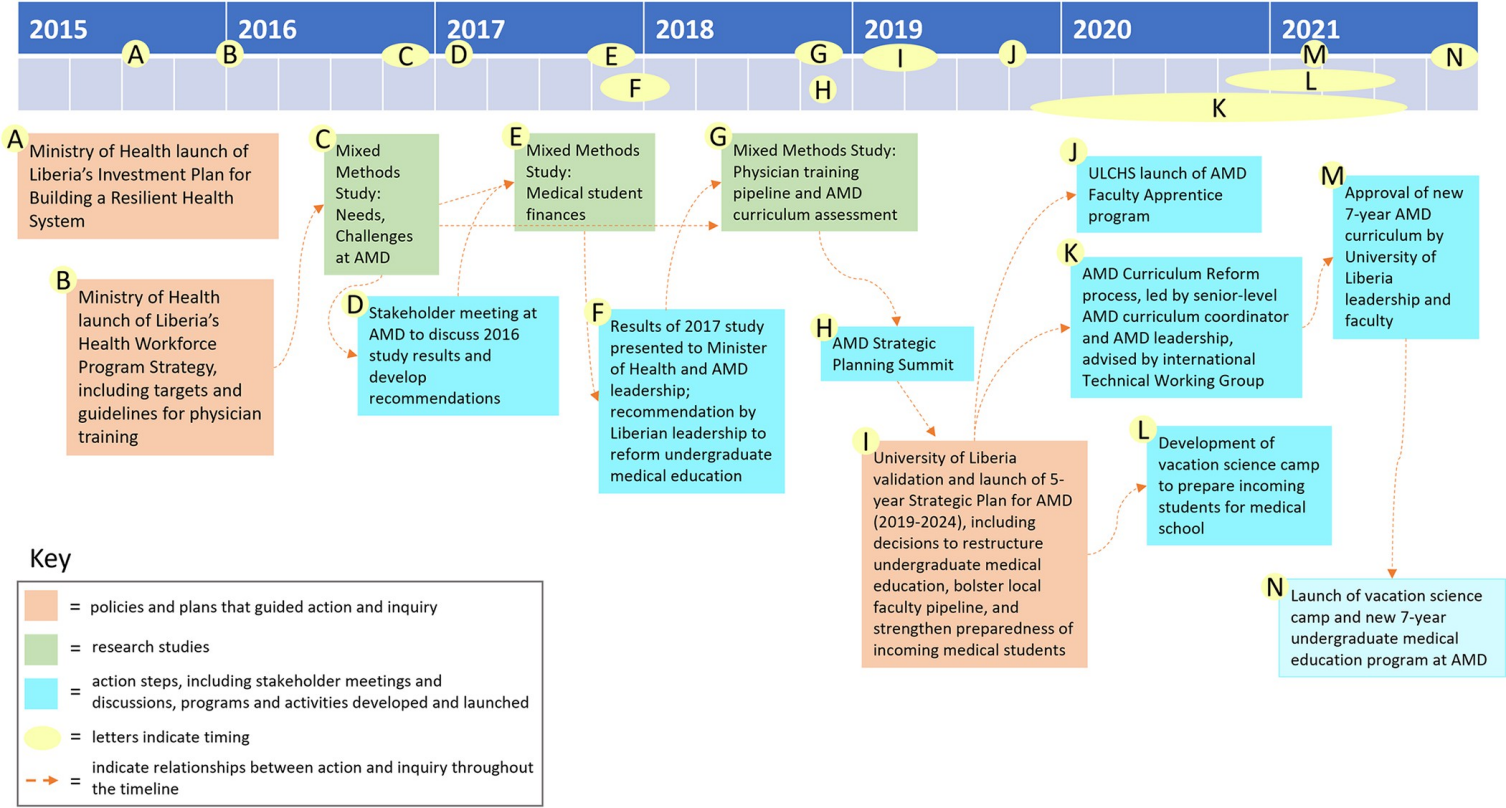
Timeline of action-inquiry to restructure medical education in Liberia.

Findings from the 2016 study were presented at a well-attended stakeholder meeting of AMD faculty, medical students, the University of Liberia President and other senior administrators, and the Minister of Health in January 2017 (Fig 4), followed by discussion. At the request of the Minister of Health, and initiating a practice that became a regular feature of the work of the community of inquiry, stakeholder input was incorporated into the final study report, resulting in consensus recommendations (Table 7) related to faculty development, curriculum, teaching, and finances to improve M.D. education in Liberia.

**Table 7:** Consensus recommendations for improving undergraduate medical education in Liberia, developed in response to 2016 study findings.

Topic	Recommendations
**Faculty**	Identify high-potential Liberian students who can serve as potential preclinical faculty
	Explore possibilities for medical residents to teach clinical topics
	Emphasize teaching experience and best practices in hiring
	Implement student evaluations beginning with the 2017–18 academic year
**Curriculum, teaching**	Review and revise the medical curriculum during the 2017–18 academic year, with attention to exam failure policy
	Allow students to view graded examinations
	Ensure that exams are consistent and fair
	Create a learning climate in which students feel comfortable asking questions of their professors
	Establish a Standing Committee on Medical and Health Education that will enable regular communication between AMD students, faculty, and administrators
**Finances**	Continue to explore AMD/UL budget, financial challenges, and mechanisms for sustainable financing of medical education

These recommendations led to development and launch of a Faculty Apprentice program for future preclinical AMD faculty to establish a sustainable pipeline of medical school faculty, given that the dearth of faculty emerged as a major challenge in the 2016 study. The Faculty Apprentice program is a four-year program that includes a blended educational program of teaching apprenticeship, mentorship and training in teaching, and graduate training in a basic science discipline in order to provide high-potential, highly motivated Liberians with both the advanced biomedical knowledge and pedagogical skills they need to become effective teaching faculty at AMD. The Faculty Apprentice program was launched in September 2019 and will graduate its first cohort of AMD junior faculty in 2023 (Fig 4). This program will be described in more detail in a forthcoming manuscript.

In order to meet the recommendation made by Liberian stakeholders related to financial data (Table 7), a second survey was conducted in 2017 to develop a more comprehensive understanding of the financial challenges faced by medical students in Liberia. The survey was administered to 83 AMD students out of 209, yielding a response rate of 39.7%. This survey provided more demographic data than the 2016 survey, indicating that AMD students had an average age of 28 (range 22–39, with half of the respondents age 30 or older). Seventy-four percent of the respondents were male and 26% were female, which was similar to the gender ratio of medical students at that time: 70% male (n = 140) and 30% female (n = 63) (S1 Fig). Fifty-four percent of respondents were preclinical students (years 1–2), with the remaining 46% representing the clinical years 3–5.

**Fig 5:**
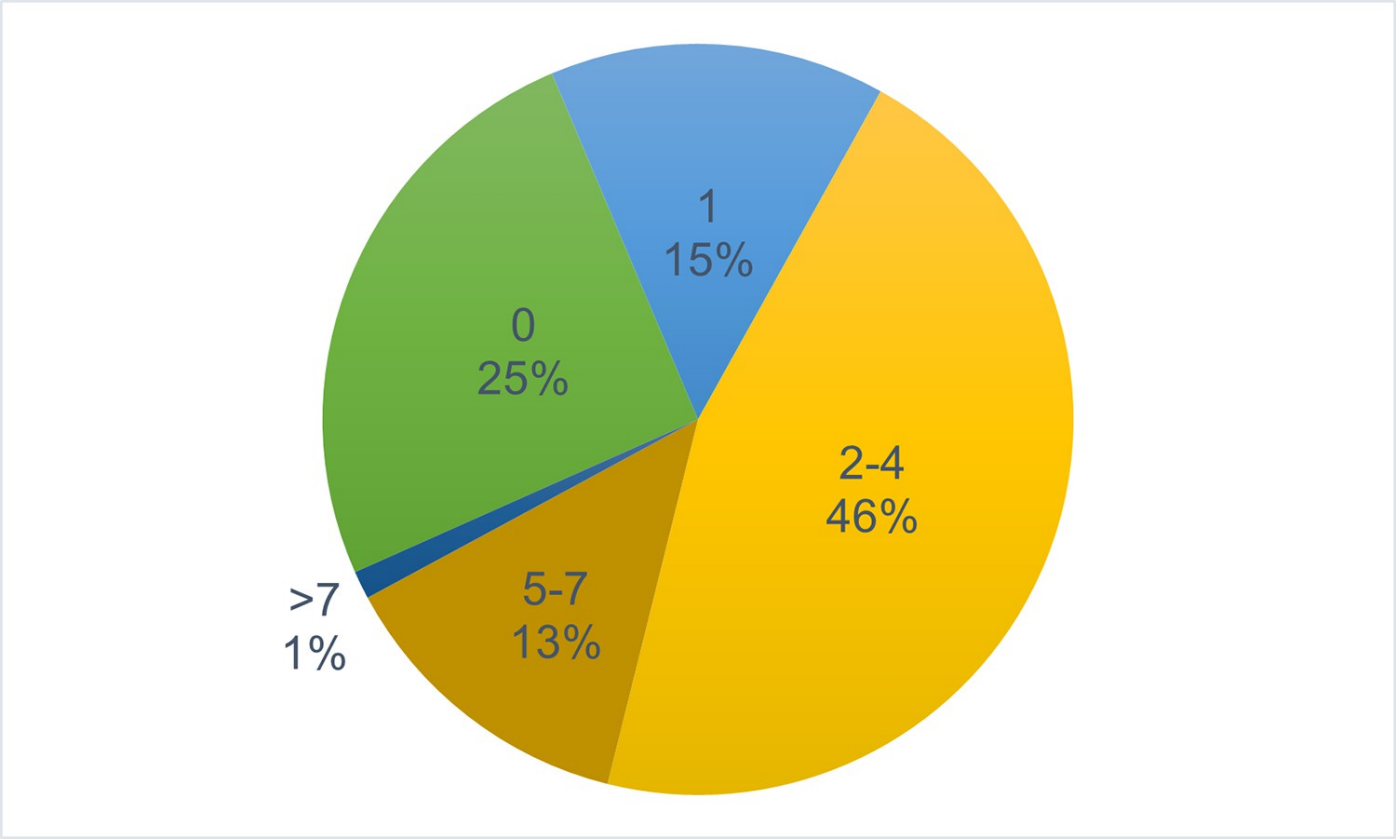
Number of financial dependents reported by AMD students (2017). This pie chart indicates the number of financial dependents reported by AMD students in 2017. Number of financial dependents ranged from 0 to more than 7. Percentage of AMD students with 0, 1, 2–4, 5–7, or more than 7 financial dependents is indicated for each category.

This survey generated more detail about AMD students’ monthly expenses, including specific costs of food, transportation, and housing as well as whether students received external financial support. When asked about financial dependents, 75% of respondents (62/83) indicated that they had at least one financial dependent (Fig 5). Forty-six percent of students (38/83) were supporting between 2 and 4 dependents, and 14% of students (12/83) reported more than 5 financial dependents (Fig 5). These data, combined with findings from 2016 that monthly government stipends were frequently not paid on time and that the majority of medical students faced significant financial difficulties and/or worried about not having enough money, added more context to the theme of financial challenges that emerged in 2016 (Tables 5 and 6). In addition, the 2017 survey indicated that 16 out of 81 students had repeated one or more year of medical education and were not on track to graduate with their matriculating class.

In stakeholder meetings following completion of the 2017 survey, Liberian members of the partnership emphasized that the existing model of M.D. training in Liberia started too late in a student’s academic career and took too long, resulting in severe financial burdens for many medical students who were trying to support their families and potentially contributing to high attrition rates. The finding that 16% of survey respondents had repeated at least one year of medical school also indicated additional financial costs for AMD, the University of Liberia, and the Government of Liberia, which fully funds medical students’ education for the duration. Liberian stakeholders also revisited concerns and recommendations expressed in response to 2016 data (Table 7) that exam failure policies may be an important root cause of delays on medical students’ progression toward their M.D. degree and/or contributing to medical student attrition.

Consensus recommendations within the community of inquiry, particularly from Liberia’s medical school and public health leadership, were that M.D. training in Liberia needed to be restructured, both to enable medical students to complete their training earlier in their careers and also to reduce the financial burdens experienced by students, the university, and the Government of Liberia. Liberian stakeholders also noted that a shorter, post-secondary school medical program would be in alignment with other medical schools in the West African region [14–17] and would revert back to the medical education structure pursued in Liberia prior to its civil war.

In 2018, building on findings from the 2016 and 2017 studies and the associated stakeholder meetings and discussions, AMD leadership called for a strategic planning process to guide and inform upcoming changes and provide a coherent strategy for both Liberian and non-Liberian partners to follow. To inform this strategic planning process, the research team developed another needs assessment of the physician training pipeline in Liberia that included survey questions related to M.D. education in Liberia. The survey was administered to 124 respondents who represented various stages along the physician-training pipeline in Liberia. Response rates for final-year medical students was 74% (27 respondents out of 37 fifth-year students). Due to the team’s lack of data on total numbers of interns, residents, and nationwide practicing physicians in 2018, response rates for those categories are not available. Of the total 124 respondents, 22% were final-year medical students, 10% were recent AMD graduates serving as clinical interns, 23% were medical residents, and 45% were physicians practicing medicine in Liberia, including medical faculty. The survey examined preparedness along various stages of the physician training pipeline, both self-assessed and assessed by others, as well as detailed course-by-course assessments of the existing preclinical and clinical AMD curriculum. The survey also explored respondents’ interests in research, teaching, and clinical specialization.

**Fig 6:**
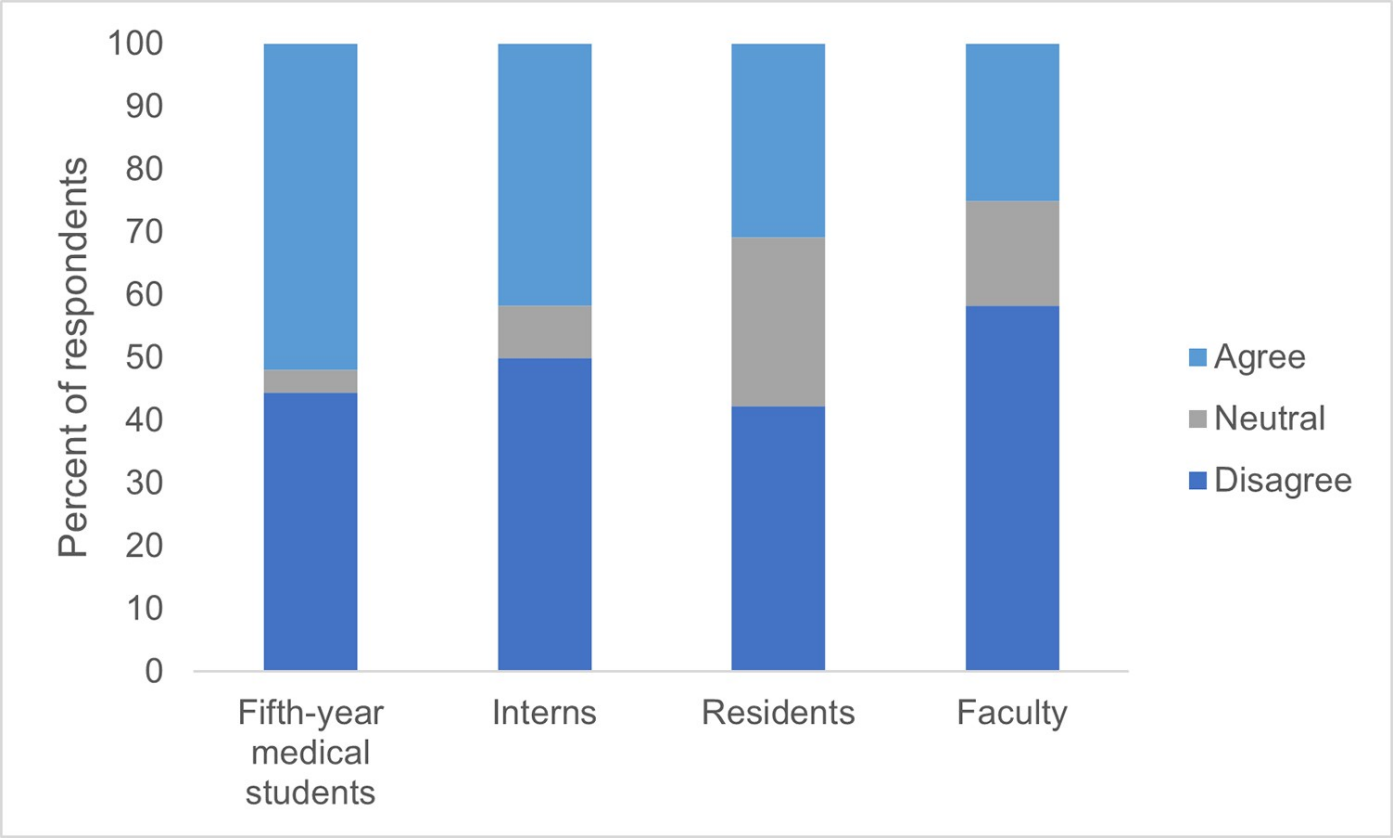
Assessment of whether incoming medical students are prepared for medical school by fifth-year medical students, medical interns, medical residents, and medical faculty.

Fig 6 shows survey respondents’ perceptions of the preparation of incoming students at AMD for performing well in the current medical school curriculum (n = 77). These data include findings from all subgroups of survey respondents. The majority of all subgroups, except for fifth-year medical students, disagreed with or felt neutral towards the statement that incoming students at AMD are fully prepared to do well in the current medical curriculum (48% of fifth-year medical students, 58% of physician interns, 69% of physician residents, and 75% of medical faculty). Notably, as the level of training and seniority of respondents increased, their agreement that incoming medical students were prepared for success decreased. These findings may indicate a Dunning-Kruger effect, which occurs when trainees at earlier stages of their careers are less able to assess their own competencies accurately than those who have progressed further in their career development [26]. In any case, it is notable that the largest percentage of respondents who agreed that incoming medical students were prepared for success was only 52%, as assessed by fifth-year medical residents, who were the least experienced in medical practice of all respondents.

**Fig 7:**
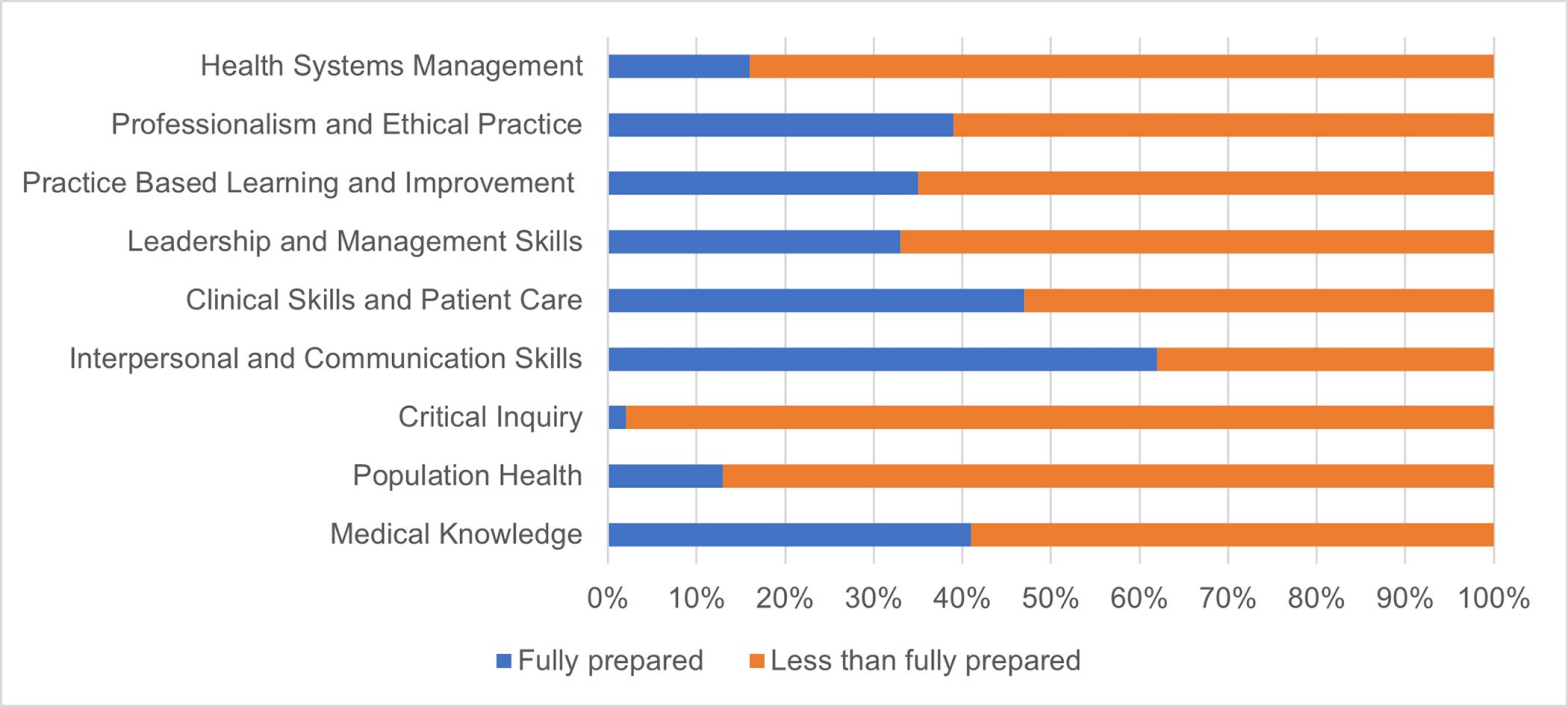
AMD graduates’ self-assessment of nine physician competencies.

When asked to self-assess on nine medical competencies adopted by other African medical schools [27], (Fig 7) AMD graduates rated themselves as less than fully prepared for health systems management (84%), professionalism and ethical practice (61%), practice based learning and improvement (65%), leadership and management skills (67%), clinical skills and patient care (53%), population health (87%), and medical knowledge (59%). Almost all Liberian-trained participants reported that they were less than fully prepared for critical inquiry (98%). The only category in which the majority of participants reported full preparation was interpersonal and communication skills (62%). Collectively, these findings indicated lack of preparedness for success and/or competency along the physician training pipeline in Liberia in 2018.

Continuing the stakeholder engagement and action-inquiry approach with an expanded partnership (Fig 1B), University of Liberia College of Health Sciences leadership presented findings from the 2018 survey first to a Grand Rounds meeting at UL’s teaching hospital with an audience that included physicians, medical faculty, medical residents and interns, international academic partners, and health care staff, and then again at a 3-day, intensive AMD Strategic Planning Summit with more than 100 participants representing the University of Liberia, the Liberian Ministries of Health and Education, funders, medical residents, interns, and students, many U.S. academic partners, and other organizations and stakeholders. The Strategic Planning Summit ultimately generated multiple recommendations and goals that were formally adopted by the University of Liberia and key medical stakeholders and leadership in Liberia in early 2019 in the form of a 5-year strategic plan (2019–2024) for the medical school (Fig 4). These recommendations included:

- restructure M.D. education in Liberia from a post-Bachelor’s program to a post-secondary, 7-year program
- adopt nine physician competencies, modeled after similar competencies utilized in other African medical schools [27]
- revise the AMD curriculum to create an integrated, competency-based curriculum, shifting away from dividing preclinical and clinical instruction, and instead incorporating both basic and clinical sciences throughout the instructional period
- develop a science camp for high-potential secondary school students in Liberia to address concerns about incoming students’ preparedness for an integrated, inquiry-based, 7-year medical school program

- establish functional teaching facilities at the university-affiliated teaching hospital
- construct additional facilities for teaching
- establish a pipeline of preclinical faculty who will be dedicated to and well-prepared for teaching in the new integrated medical curriculum through the AMD Faculty Apprentice Program
- develop and implement a financial sustainability plan
- establish human resource and financial management capacities at the medical school, including grant management

Following the recommendations of the 2019–2024 AMD Strategic Plan, a new 7-year medical curriculum was developed by AMD faculty with active participation and support from many members of the community of inquiry, who collectively formed a working group with international technical advisors working alongside the local faculty members. The AMD Curriculum Working Group met weekly from the end of 2019 through 2021 to develop and plan implementation of the new, 7-year integrated curriculum for AMD. This curriculum was approved first by the University of Liberia leadership Cabinet, then by the Faculty Senate in March 2021, then was officially launched in November 2021, with full implementation in 2022 (Fig 4).

Continuing its action-inquiry, collaborative approach, the community of inquiry also launched an inquiry-based, STEM preparatory camp for 11^th^- and 12^th^-grade secondary school students in 2021 to help address the lack of preparedness of incoming medical students indicated by the 2018 study (Fig 6). In its second iteration in 2022, the camp, called Camp xSEL, for “eXcellence in Science Education for Liberia” included students from all 15 counties Liberia to ensure opportunities for both urban and the traditionally underserved rural students, and it also included 51% females to help improve gender parity in medical education in Liberia. Also in 2021, members of this community of inquiry received a 5-year, $15 million USAID grant to strengthen research utilization at the University of Liberia College of Health Sciences. This award has supported establishment of a Center for Teaching, Learning, and Innovation, launched in 2022, as well as many educational programs, including continuation of the Faculty Apprentice program and Camp xSEL. Additional establishment of human resources, financial, and grant management offices for ULCHS has also been supported through this new award. The members of the community of inquiry applied for the USAID award together, further extending their collaborative ecological inquiry approach to organizational transformation described here.

## Discussion

This paper reports on the development of an international “community of inquiry” [18, 19] whose members have paired inquiry in the form of multiple research studies, and action in the form of numerous stakeholder meetings, planning meetings, and curriculum development meetings, both in person and remote, from 2015 to the present to transform medical education in Liberia. The community of inquiry includes a few key, longstanding partners from Liberia and grant-funded partners from the U.S. who have an ongoing close, trusting working relationship, as well as many individuals who temporarily joined the project and made key contributions, including faculty from Liberia and other countries in West and East Africa, and students and research assistants from Liberia and the U.S (Fig 1). The community of inquiry has utilized knowledge gained in an ongoing learning process to inform transformation of M.D. education in Liberia, as well as many other capacity-building initiatives in the physician and health workforce training pipeline.

### Keys to success

The development of the community of inquiry, and indeed the transformational work of this community, was not foreseen by its members, nor guaranteed at the outset. Rather, evoking the process of collaborative ecological inquiry described by Torbert [18, 19], the work and learning activities of this community evolved and expanded through the continual investment of the partners in building and maintaining trusting relationships and in mutual, ongoing efforts to pair inquiry with action. Members of the community of inquiry were supported by different, often distinct, funding sources throughout this partnership (Fig 1). Many individuals involved in this work devoted extra and/or unpaid hours to these activities during periods when funding was inadequate. Crucially, the research aims and plans for action described here were envisioned and directed by the Liberian partners and supported by US-based and regional foreign partners from West Africa and Ethiopia. Further, building trust with the broader AMD community, which formed the participant base for all of the assessments, was essential. The same team that conducted the research was also simultaneously designing and implementing changes, which created a responsive relationship between the research-practitioners and the stakeholders (including AMD students, AMD faculty, both Liberian and foreign faculty from the West African region and Ethiopia, medical school leadership, and medical practitioners) and broadened the community of inquiry to include the community of participants. These relationships were central to the generation of evidence and recommendations for action, as well as ongoing implementation. The community of inquiry approach also ensured that all research conducted by the partnership followed, rather than led, the decisions, priorities, and goals of partners in Liberia.

### Limitations and challenges

Medical faculty perspectives were underrepresented in the studies conducted in 2016 and 2017. This was because AMD had only 3 full-time Liberian faculty members during that period, all of whom also served in administrative roles (Dean, Assistant Dean/Clinical Coordinator, and Preclinical Coordinator). The remaining medical faculty at AMD also held other—sometimes multiple—full-time jobs, often as clinical care providers and high-level hospital and/or government administrators. Thus, the first two assessments reported here primarily reflected medical students’ perspectives and experiences. Liberian participants in the 2016 and 2017 research evaluation teams helped non-Liberian researchers understand that faculty shortages, low faculty salaries, and lack of professional development opportunities for medical and health sciences faculty contributed not only to the challenges reported by medical students, but also to the difficulty of obtaining faculty members’ participation.

Response rates in both the 2016 and 2017 studies were 27.9% and 39.7%, respectively. In 2016, surveys were administered during a school break, and many students were not on campus. Although the survey was distributed both on paper and digitally in 2016, few responses were received via the digital survey form, leading to a lower-than-expected response rate. In 2017, the survey was administered on paper only, and multiple attempts were made to request input by the AMD student research assistant in the group, leading to a higher response rate than in 2016. The percentage of female respondents was slightly lower among 2017 survey respondents than in the medical school population (25% vs 30%, respectively), suggesting that the input of female medical students related to financial data may have been underrepresented. Response rates for the 2018 study were only partially assessed due to lack of total data on actual numbers of interns, residents, and practicing physicians nationwide at that time.

Due to difficulty locating/contacting former AMD students, none of the three assessments included the perspectives of students who failed or left medical school prior to graduation. As a result, the data presented may not comprehensively capture the unmet needs of all students who ever enrolled at AMD, and no causal relationships could be established between learning barriers at AMD and medical students’ failing or dropping out. In addition, the research team was not able to obtain details about failure rates or reasons for failing out of school at AMD, as none of the surveys probed this topic, and the medical school registrar’s office did not track detailed exit information about students who departed the medical school prior to graduating.

In the 2018 study, the perspectives of medical care providers, students, and trainees from the rural areas of the country were not well-represented. Despite multiple outreach efforts to more rural practitioner groups, survey respondents primarily practiced medicine in the densely-populated urban area of the country (i.e. the capital city of Monrovia and the surrounding Montserrado county). This is an unfortunate omission, especially given that rural areas and health needs are often underrepresented in Liberian health data. We feel, however, that this limitation does not negate the value of that survey data, both because medical education (before and after students obtain their M.D. degree) is anchored in and around Monrovia, since AMD is the only medical school, and the only teaching hospital is also located in Monrovia. In addition, many of the survey respondents were AMD alumni and medical faculty or physician consultants who play a role in medical education in Liberia.

### Insights from this work

Several insights have emerged from the work of this partnership that may be applicable to similar work strengthening medical education and/or health workforce systems in resource-constrained settings:

1. The studies conducted in 2016–18 highlighted the need for sustained resource investment in faculty pipeline development and other educational programs that can be executed and maintained by local higher educational institutions.
2. Workforce development programming, more generally, must be anchored in permanent, local institutions—and not offered on an *ad hoc* basis tied to a certain funding source or funding period—in order to be sustainable. The community of inquiry, at the direction of medical and health leadership in Liberia, have developed faculty development programs, a faculty apprentice program, and Camp xSEL, a vacation science camp for future premedical students, that are anchored in and administered by faculty and staff at the University of Liberia College of Health Sciences.
3. The research teams that conducted the needs assessments from 2016–2018 included undergraduate and medical students from U.S. and Liberian academic institutions (Yale, Vanderbilt, and ULCHS), some of whom were participating in capstone research courses, with close supervision and guidance from U.S. and Liberian faculty. This approach to action-inquiry through teaching and mentorship contributed to self-reflection and engagement across the community of inquiry.
4. The partnership’s ongoing emphasis on preserving trust, maintaining honesty (even when difficult), and following the vision, goals, and priorities of leadership in Liberia has contributed to the creation of a unique and highly successful community of inquiry that has persisted over multiple years, through a variety of funding sources, and across institutions, cultures, and countries.
5. The participation of individuals from different countries (Liberia and the U.S.) representing multiple different perspectives was essential for this community of inquiry to form and work effectively. The Liberian research team members’ input provided crucial, otherwise missing, data to facilitate improved understanding and inquiry-based actions that could build meaningfully and appropriately upon study results. For example, researchers from the U.S. learned from Liberian researchers throughout this period that many medical faculty members teach at the medical school despite their personal time constraints and lack of training in teaching methods out of a sense of duty to ensure that the school remains open. In addition, the outsider perspectives and experiences provided by the U.S.-based partners enabled the introduction of new ideas, approaches, and teaching resources that had previously been utilized in their own, high-resource settings, which the partnership then adapted to fit the Liberian context. These included the introduction of team-based learning as a teaching approach to improve student-centered learning with low numbers of faculty [28], faculty development workshops to provide pedagogical skill-building for existing AMD faculty members, and strategies for developing an integrated M.D. curriculum as part of the overall transformation of AMD.
6. The members of the international community of inquiry described here are committed to equitable, decolonized global health work. For us, this meant waiting to present the results of the 2016–18 studies as part of a comprehensive, contextualized story including their impact on transforming medical education in Liberia. We felt that the converse approach, reporting data from individual studies, stripped of context and impact, could have generated a more extractive, “helicopter research” [29, 30] outcome, inaccurately elevating the role played by non-Liberian members of this community of inquiry without equitably representing the crucial roles played by Liberian members of this group. We recognize and acknowledge that presenting multiple data-generating studies as part of a larger action-inquiry approach to organizational transformation is unusual, and represents a novel approach to reporting global health “research,” per se. We suggest, however, that in this international community of inquiry, as described by Torbert, "researchers…are not disembodied from the communities they are researching. Nor are they merely participant observers, present on the scene of action…Instead they are observant participants seeking to join with the other participants in creating a community of inquiry where all are both participating and inquiring.” [18]

Finally, it is worth noting that the emphasis by Liberian leadership on building and strengthening sustainable institutions and systems has been an ongoing effort by health leadership in Liberia prior to and throughout the 2014–15 Ebola epidemic, which has continued throughout the COVID-19 pandemic. Although requiring immediate responses and resources, the Ebola and COVID epidemics highlighted the ongoing need for strengthened health and educational systems in Liberia (and globally). The experience of this community of inquiry emphasizes the importance of long-term commitment to and investment in health systems strengthening, before, during, and after short-term responses to health crises, as well as the requirement for visionary leadership in Liberia to set the path for the whole group to follow.

## Conclusion

We report here a multiyear action-inquiry process to understand and address challenges in medical education in Liberia, culminating in organizational transformation of the country’s only medical school, A.M. Dogliotti School of Medicine. Informed by data collection and analysis, this work is founded and nurtured through a trusting, mutual-learning partnership, in which leaders in Liberia set the vision and guide the work, with help, input, research expertise, and intentional followership [31] from U.S.-based academic researchers. Through an approach described in the social science literature as collaborative ecological inquiry, in which learning, action, self-reflection, multiple perspectives, and shared purpose are combined and collectively employed toward peaceful personal and organizational change [18], this international team of researcher-practitioners has formed a community of inquiry and developed a robust, evidence-based approach to transforming M.D. education in Liberia. We recommend the approach described here as a model that could be valuable for others seeking to form sustainable global health partnerships that lead to organizational transformation.

## Supporting information

S1 Fig Student Demographics at AMD, 2016–18. (TIF)

S1 Table Actual vs Needed Number of Faculty at AMD (2016). (TIF)
