# Supplementary figures and images for: Transforming medical education in Liberia through an international community of inquiry
Source: PLOS Glob Public Health. 2023 Mar 8;3(3):e0001610. doi: 10.1371/journal.pgph.0001610 (PMC10021565; doi:10.1371/journal.pgph.0001610)

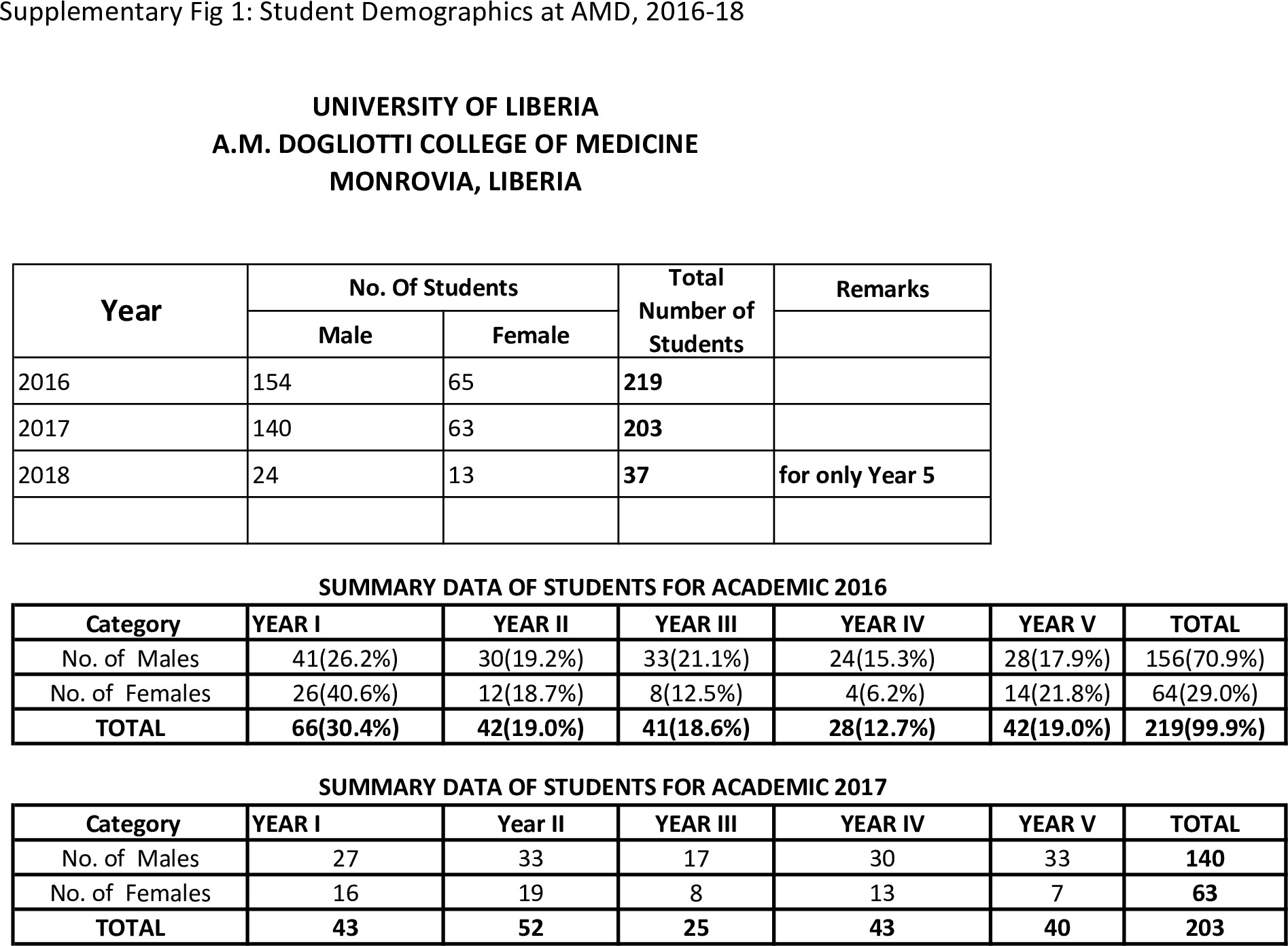

Supplement: S1 Fig — (TIF) [file pgph.0001610.s001.tif]

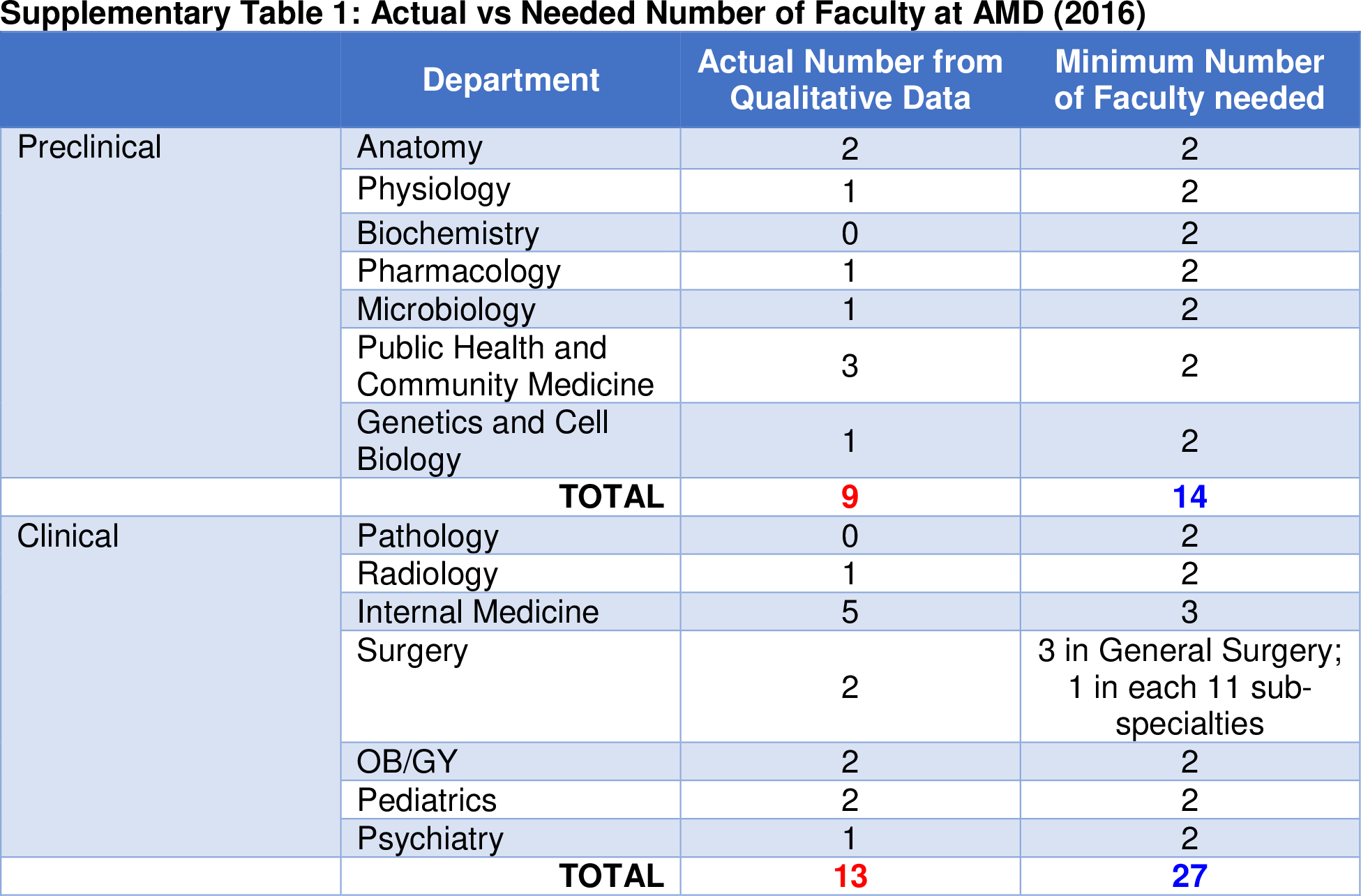

Supplement: S1 Table — (TIF) [file pgph.0001610.s002.tif]
